# Fluorescent Guided Surgery in the Surgical Management of Glioma: The Dawn of a New Era

**DOI:** 10.3390/brainsci10040237

**Published:** 2020-04-16

**Authors:** Kostas N. Fountas

**Affiliations:** Department of Neurosurgery, Faculty of Medicine, University of Thessaly, Biopolis, 41110 Larisa, Greece; fountas@uth.gr

A growing body of evidence supports the importance of marginal or even supramarginal resection in cases of high- but also of low-grade gliomas [1,2]. Several clinical series have clearly demonstrated that a more radical resection provides better overall survival, progression-free survival, as well as better quality of life for patients with high-grade gliomas [1,2,3]. Moreover, the incidence of procedure-associated complications is significantly lower in cases of perilesional glioma resection [4]. Indeed, from all the identified factors affecting the survival and the overall outcome of patients with glioma, the only one that can be altered is the extent of resection, since the patient’s age, or the glioma’s molecular signature cannot be influenced.

Many strategies have been recently developed for maximizing the extent of resection. The wide employment of neuro-navigation, the utilization of intraoperative imaging (ultrasound and/or MRI), the exponential increase in the application of intraoperative direct electrical stimulation and cortical and subcortical mapping techniques via an awake craniotomy, as well as the usage of fluorescent guided surgery have provided the opportunity to maximize the extent of resection in glioma cases, while at the same time, the patients’ safety and their functional neurological status remain intact.

Maugeri et al. [5] accurately outlined in their current review study the emerging role of fluorescent-guided surgery in the management of patients with high-grade gliomas (HGGs). They present in a concise way, their analysis results regarding the clinical safety and efficacy, as well as the accuracy of fluorescent-guided surgery. They have systematically examined the efficacy of 5-ALA in HGG resection, and its impact in the overall outcome of these patients. Likewise, they have meticulously explored the clinical safety and the efficacy of Fluorescein Sodium (FS) in the surgical resection of HGGs. The authors have adequately identified several issues associated with the overall cost of fluorescent-guided surgery, and its financial impact on the European and North American health systems, especially during the era of the upcoming silver tsunami due to the exponential increase of geriatric populations, and the associated increased incidence of glioma cases. 

The authors in their in-depth analysis also examine the future perspectives of fluorescent-guided surgery (FGS), identifying the necessity for performing randomized controlled studies for further evaluating the exact accuracy of FS [5]. They also elaborate on the potential of developing other fluorescent substances, which may identify different cellular patterns or unique metabolic characteristics of cancerous glial cells, targeting thus exclusively malignant cells, and outlining the actual margins of diffuse, non-enhancing gliomas.

Special emphasis needs to be given to the potential role of FGS in the management of patients with recurrent glioblastoma multiforme (GBM) [6,7,8,9,10,11,12,13,14,15]. Della Pupa et al. [8] reported that a gross total resection was accomplished (GTR) in 94% of their cases. Similarly, Archavlis et al. [9] postulated that GTR was achieved in 59% of their cases by employing FGS. However, the employment of FGS in the surgical resection of recurrent HGGs resulted in a slightly increased incidence of new postoperative neurological deficits in one series [10]. Contrariwise, Stummer et al. [11] in their multicentric surgical series found no increased incidence of procedure-associated complications. It needs to be pointed out that the reported false positive rate of FGS in the cases of recurrent HGG resection ranged between 42%–45% [12,13,15]. On the other hand, other series reported a quite low false positive rate in recurrent GBM surgical series [14]. A recent review study identified that the incidence of false positive results is higher in recurrent HGGs however there is strong evidence that FGS has a high positive predictive value [15]. It also needs to be noted that there are very few clinical series indicating that the employment of FGS resulted in longer overall survival [9]. However, there was no statistically significant difference in the progression-free survival in the group of patients undergoing FGS for their recurrent GBM resection [7].

Another subgroup of glioma cases demonstrating special interest is that of patients with low-grade gliomas (LGGs) [16,17,18,19,20]. The potential role of FGS in the resection of LGGs remains to be defined. It is generally accepted that in these LGGs, in which there is no contrast enhancement on the conventional MRI, there is no uptake of the administered currently used fluorescent agents [16,17,18,19]. Therefore the employment of FGS provides no benefit in these cases. Indeed, there are a few published series which have failed to demonstrate any advantages of using FGS in the surgical management of non-enhancing LGG patients [16,17,18,19]. 

The development of novel fluorescent substances, which may target LGG cells could provide the opportunity for a more extensive resection, especially in diffuse, non-enhancing gliomas. Additionally, the importance of combining the method of fluorescent surgery along with all other developed and emerging surgical strategies for maximizing glioma resection cannot be overemphasized. The combination of FGS and intraoperative cortical/subcortical stimulation and mapping might further facilitate the goal of supramarginal resection in LGG cases, and thus improve the patient’s overall outcome. Moreover, the employment of FGS along with intraoperative Raman spectroscopy may even identify metabolically active areas, which cannot be visualized by the conventional preoperative imaging studies [21]. Nevertheless, it has to be kept in mind that FGS is a complementary to all other modalities, which can facilitate the extirpation of a glioma without compromising the patient’s neurological status.

Finally, the issue of the overall cost of FGS needs to be seriously taken into consideration. In an era in which health expenses are constantly expanding and most health systems struggle, emerging technologies need to be of low cost in order to be easily accessible and widely available. The cost of each of these adjuvant methodologies has to be counterweighted by its positive effect on the patient’s survival and overall outcome, as well as the long-term, associated health expenditures. 
